# A Toddler With Iron Deficiency Anemia Secondary to a Yolk Sac Tumor of the Stomach: A Case Report and Literature Review

**DOI:** 10.1155/crpe/9954122

**Published:** 2026-06-22

**Authors:** Nabil Saleem, Pamela B. Sylvestre, Franklin L. Chien, Raul S. Gonzalez, Jose E. Velázquez Vega, Sarah Mitchell

**Affiliations:** ^1^ School of Medicine, Emory University, Atlanta, Georgia, USA, emory.edu; ^2^ Aflac Cancer and Blood Disorders Center, Children’s Healthcare of Atlanta, Atlanta, Georgia, USA, choa.org; ^3^ Cincinnati Children’s Hospital Medical Center, Cincinnati, Ohio, USA, cincinnatichildrens.org; ^4^ Department of Pathology, Children’s Healthcare of Atlanta, Atlanta, Georgia, USA, choa.org

**Keywords:** anemia, endodermal sinus tumor, gastric solid tumor, germ cell tumor, pediatric hematology/oncology, yolk sac tumor

## Abstract

Yolk sac tumors, a subset of malignant germ cell tumors originating from gonadal germ cells, most often present as ovarian or testicular tumors in adolescents, sometimes in combination with different types of histology as a mixed malignant germ cell tumor. We describe a female toddler presenting with severe iron deficiency anemia who was found to have a gastric pure yolk sac tumor. Pure yolk sac tumor of the stomach has rarely been reported and is even less frequent in the pediatric population. Our review of the medical literature found 12 case reports of gastric pure yolk sac tumor, of which only 1 was in a child, a 3‐year‐old male. Our patient underwent subtotal gastrectomy and received adjuvant systemic chemotherapy and is tumor‐free at 3 years following surgery. This is the first reported case of a gastric pure yolk sac tumor in a pediatric female, brought to clinical attention due to significant gastrointestinal blood loss.

## 1. Introduction

Anemia is a common laboratory finding in many toddler‐aged patients, with incidence approaching 20% for children between the ages of 0 and 4 [1]. It is most commonly due to iron deficiency anemia secondary to inadequate iron intake and often associated with excessive cow’s milk intake. Dietary modifications and iron supplementation typically lead to quick resolution of anemia. However, anemia refractory to treatment or severe anemia may be related to rare etiologies, such as malignancies.

Germ cell tumors (GCTs) are rare in the pediatric population, accounting for 3% of pediatric malignancies in patients aged 0–18 years. As a result, there is a paucity of literature describing these malignancies. Incidence appears to rise in older adolescent patients, with GCTs making up 15% of malignancies diagnosed during adolescence [2]. In both adult and pediatric patients, GCTs are thought to arise from germ cells in the early stages of development and, therefore, typically present in gonadal locations. GCTs can be homogeneous, such as a pure yolk sac tumor (YST), which is more common in children. The histology can also be a mixture of multiple subtypes to form mixed GCTs, which are more common in adults [3]. Due to the rarity of pediatric GCTs, the variable locations they can present in, as well as the complex histology underlying these malignancies, diagnosis can be challenging. Contemporary diagnosis of GCTs relies on clinical history, physical examination, serum tumor markers, immunohistochemical stains, and histopathology [4]. We report an unusual case of a gastric pure YST in a female toddler who presented with severe iron deficiency anemia secondary to gastrointestinal blood loss.

## 2. Case Presentation

A 23‐month‐old Caucasian female was brought to the emergency department by her parents following a loss of consciousness with a history of progressive fatigue and pallor. Review of systems for other symptoms was negative, including nausea/vomiting, abdominal pain, and weight loss. She was tachycardic (heart rate of 157 bpm) and pale in appearance. She had no tenderness to palpation of the abdomen, and physical examination was otherwise unremarkable. Laboratory analysis was notable for severe anemia with a hemoglobin of 2.5 g/dL, an elevated reticulocyte percentage of 7.6%, normocytic red blood cells, and normal counts of white blood cells and platelets. A complete metabolic panel and hemolysis markers were reassuring (Table 1). The fecal occult blood test was positive, though sample was obtained via digital rectal examination that could have caused a false‐positive result. Iron studies revealed a serum ferritin of < 1 ng/mL and low iron saturation of 2% with normal total iron‐binding capacity of 326 μg/dL, collectively consistent with iron deficiency (Table 1). Birth history was essentially unremarkable; the patient was born weighing 2.92 kg at 37 4/7 weeks’ gestation by cesarean section due to breech position. Past medical history was only significant for left developmental hip dysplasia, successfully treated with a harness. Family history was negative for any malignancy on either mother or father’s side of the family, and the mother’s history is notable for iron deficiency anemia during pregnancy.

**TABLE 1:** Key laboratory values at time of initial hospitalization, repeat hospitalization, and annual follow‐up evaluations.

Test	Result	Units	Reference range
*Initial presentation*			
Hemoglobin	2.5	g/dL	11.5–13.5
Mean corpuscular volume	71.3	femtoliters	70–86
Red cell distribution width	19.9	%	11.5–14.5
White blood cell count	12.54	thousand/μL	6.0–17.0
Platelet	427	thousand/μL	150–450
Alanine aminotransferase	38	U/L	11–30
Reticulocyte percentage	7.6	%	0.9–2
Lactate dehydrogenase	212	U/L	192–321
Direct Coombs test	Negative		Negative
Serum iron	8	μg/dL	30–150
Iron saturation	2	%	(None reported)
Ferritin	< 1	ng/mL	10–99.99
Fecal calprotectin	68	μg/g	≤ 49

*Repeat hospitalization (5 days after initial presentation)*			
Hemoglobin	4.3	g/dL	11.5–13.5
Serum alpha fetoprotein	11,543	ng/mL	1–11
beta‐hCG	< 5	mIU/L	< 5

*Evaluation at 1-year follow-up*			
Hemoglobin	12.0	g/dL	11.5–13.5
Serum alpha fetoprotein	< 2	ng/mL	1–11
beta‐hCG	< 2.42	mIU/L	< 5

*Evaluation at 2-year follow-up*			
Hemoglobin	11.6	g/dL	11.5–13.5
Serum alpha fetoprotein	< 2	ng/mL	1–11
beta‐hCG	< 2.42	mIU/L	< 5

*Evaluation at 3-year follow-up*			
Hemoglobin	12.2	g/dL	11.5–13.5
Serum alpha fetoprotein	< 2	ng/mL	1–11
beta‐hCG	< 2.42	mIU/L	< 5

The patient was admitted to the intensive care unit and transfused slowly with three aliquots of 5 mL/kg packed red blood cells. Upon further discussion, parents clarified the patient’s diet as finger foods, including fruits, vegetables, and breastmilk until 18 months of age followed by a transition to whole cow’s milk, typically drinking 12–16 oz daily. In the weeks preceding her admission, her family had recently added meat and iron‐rich grains to her diet per her pediatrician. Dark and/or grossly bloody stools were denied.

The patient was started on 5 mg/kg of oral iron supplementation for a presumed diagnosis of iron deficiency anemia secondary to inadequate dietary intake. Nutrition recommendations regarding increased dietary iron and restriction of cow’s milk intake were provided. Transient erythroblastopenia of childhood (TEC) as well as viral marrow suppression were considered, potentially compounding underlying iron deficiency in this patient. Parvovirus immunoglobulins were drawn and later returned negative.

On hospital day #2, the patient developed dark stools, attributed to the enteral iron supplementation. Fecal occult blood testing was again positive, though it was presumed to be a false positive in the setting of enteral iron supplementation. The gastroenterology service was consulted. Additional studies were performed including a technetium‐99 m pertechnetate radionuclide study (Meckel’s scan) to evaluate for Meckel’s diverticulum and a right upper quadrant ultrasound to evaluate for a duplication cyst; both studies were normal. Fecal calprotectin was reassuring against inflammatory bowel disease. Cross‐sectional imaging and endoscopic evaluation were deferred. Given clinical improvement, the patient was discharged home with close outpatient gastroenterology and hematology follow‐up.

**FIGURE 1:**
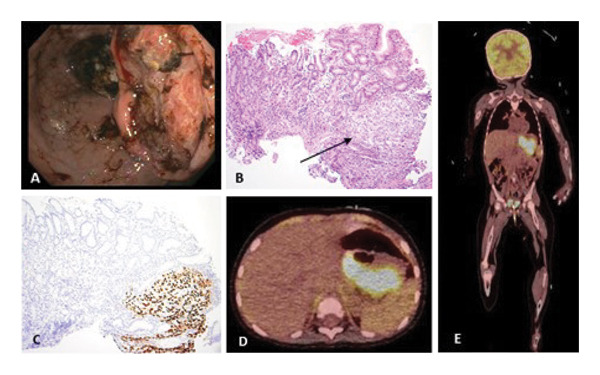
(A): Endoscopic view of a large, ulcerated mass on the greater curvature of the stomach with oozing, bleeding, and stigmata of recent bleeding. (B): Gastric mass biopsy containing a small portion of tumor (arrow), photographed at 40*x* magnification. (C): Immunohistochemical stain for SALL4 showing strong positivity in tumor cells, characteristic of germ cell tumors (photographed at 40*x* magnification). (D & E): FDG‐PET/CT scan illuminating the gastric mass without evidence of metastasis.

Two days after hospital discharge, the patient presented to her pediatrician with recurrent lethargy and pallor. Laboratory work confirmed recurrent severe anemia (hemoglobin 4.3 g/dL), and parent history was concerning for a large, loose, black stool. She was again evaluated in the emergency department and admitted to the intensive care unit, this time tachycardic (heart rate 170 bpm) and tachypneic (respiratory rate to the high 30 s). Blood pressure was normal. She was transfused slowly with two aliquots of 5 mL/kg packed red blood cells, with improvement in hemoglobin to 7.5 g/dL. Due to melanotic stools and concern for gastrointestinal blood loss, MRI of the abdomen and pelvis was obtained and revealed marked gastric wall thickening and a nodular mural mass along the posterior gastric body. Esophagogastroduodenoscopy (EGD) confirmed a large, ulcerated mass seen along the greater curvature of the stomach with “oozing bleeding” (Figure 1, A). Endoscopic biopsy provided a small sample of tumor, yielding a diagnosis of YST (Figure 1, B & C). Colonoscopy was normal. Serology studies revealed markedly elevated serum alpha‐fetoprotein (AFP) of 11,543 ng/mL and undetectable beta human chorionic gonadotropin (beta‐hCG).

**FIGURE 2:**
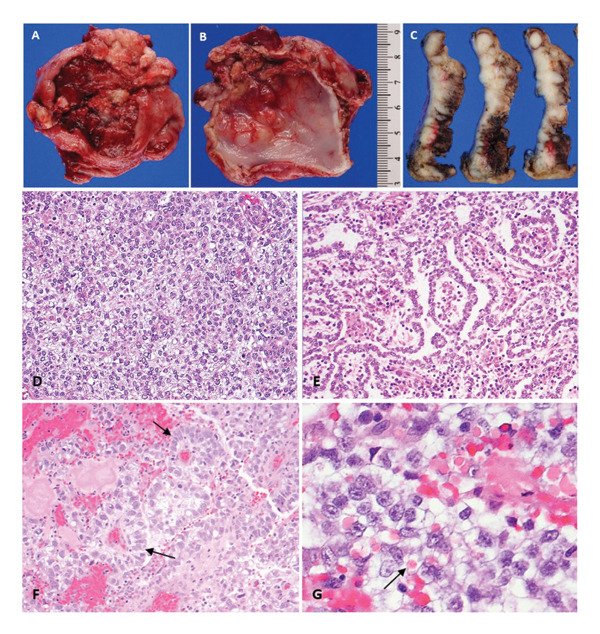
(A): Luminal aspect of the resected gastric mass with irregular and ulcerated luminal surface. (B): Serosal aspect of the resected gastric mass with nodular protrusions. (C): Cross section of the resected gastric mass (post fixation) depicting marked gastric wall thickening by the mural neoplasm. (D): Low‐power microscopic view of an area of tumor with solid growth pattern (photographed at 100*x* magnification). (E): Low‐power microscopic view of an area of tumor with papillary growth pattern (photographed at 100*x* magnification). (F): Medium‐power microscopic view of tumor with Schiller–Duval bodies (arrows), photographed at 200*x* magnification. (G): High‐power microscopic view of tumor with intracellular hyaline globules (arrow), a classic feature of yolk sac tumors (photographed at 400*x* magnification).

Staging evaluation via fluorodeoxyglucose positron emission tomography (FDG‐PET) scan revealed a hypermetabolic gastric mass and no other areas of FDG avidity to suggest metastasis (Figure 1, D & E). The patient underwent a subtotal gastrectomy. The specimen was a discoid portion of stomach, measuring 6.9 × 5.6 × 2.1 cm with a large, centrally ulcerated 6.9 × 4.7 × 1.5 cm mass occupying most of the specimen and grossly abutting the surgical margins (Figure 2, A). The surgical margins were negative but close, coming within 0.1 cm of the tumor. No lymphovascular invasion nor perineural invasion was identified. No tumor necrosis was identified. The serosa was partially multinodular (Figure 2, B). Serial sectioning revealed a transmural, tan‐white mass (Figure 2, C). As a result of serosal invasion of the visceral peritoneum, the tumor was staged as pT4a. Microscopically, the tumor had both solid and papillary growth patterns (Figure 2, D & E). Schiller–Duval bodies (Figure 2, F) and intracellular hyaline globules (Figure 2, G), both classic features of YST, were present. By immunohistochemistry, the tumor cells were positive for SALL4 and AFP, focally positive for glypican‐3 and EMA, and negative for OCT3/4 and cytokeratin 7. This immunoprofile, in conjunction with the histomorphologic features, supported the diagnosis of YST. No other GCT components nor an adenocarcinomatous component were identified. Following surgery, the patient received four cycles of adjuvant systemic chemotherapy with bleomycin, etoposide, and cisplatin, a common treatment regimen for YSTs [5]. AFP level normalized by 3 weeks after the start of chemotherapy. At last follow‐up 3 years postgastrectomy, she continues to remain in good health without evidence of tumor recurrence by imaging and with normal AFP and undetectable beta‐hCG levels.

## 3. Discussion

Abdominal masses in infants and toddlers can go unnoticed for quite some time, given that this patient population cannot reliably relay associated symptoms. Additionally, symptoms can be nonspecific, such as abdominal distension or mild abdominal pain, both of which are often seen with constipation. Other nonspecific symptoms that may lead to detection of an abdominal mass include vomiting, hypertension, and gastrointestinal tract bleeding. Not uncommonly, palpation of an abdominal mass is the first sign of an abdominal solid tumor in infants and toddlers.

In the neonatal/infantile population, abdominal masses occur in approximately 1 out of 1000 births and are typically benign [6]. Examples include benign renal pathologies such as hydronephrosis or renal cysts, as well as duplication cysts and a “pyloric olive” (palpable hypertrophied pylorus associated with pyloric stenosis) [7]. Malignant abdominal tumors in infants (< 1 year of age) and toddlers (1–4 years of age) are rare, dominated by neuroblastoma (the most common malignant abdominal tumor in infants), Wilms tumor (the most common renal malignancy in children), and hepatoblastoma (the most common primary malignant liver tumor in children) [7].

YSTs (also known as endodermal sinus tumors) are a type of GCTs originating from gonadal germ cells. They can occur as pure YSTs or as a component of mixed GCTs, with pure YSTs more likely to occur in children and mixed GCTs with a YST component more likely to occur in older patients [8]. The incidence of prepubertal, pure YST in children has been reported to be 2–3 cases per million children per year up until Age 6, making this an exceedingly rare tumor in children [9]. Gonadal presentations are most common, though various extragonadal sites have been reported [3, 10–14]. In female patients, one‐third of YSTs on average are extragonadal [3]. The male incidence of extragonadal YST is not known; however, pure YST of the testis is the most common testicular neoplasm in male children [15]. Most extragonadal GCTs arise in midline sites (such as mediastinum, retroperitoneum, sacrococcygeal area, and pineal/suprasellar region of the brain), attributed to incomplete migration of primordial germ cells from the primitive streak to the gonadal ridge during embryogenesis [16]. With the stomach not in line with the migration path of primordial germ cells, the etiology of gastric GCTs remains an enigma. One hypothesis is that gastric GCTs may arise in the stomach from aberrant migration of germ cells [17]. Another hypothesis is that gastric GCTs may arise in the stomach from seeding of intravascular circulating germ cells. Because some adenocarcinomas of the stomach may be accompanied by GCT components, some authors have proposed that retrodifferentiation of adenocarcinoma may result in the development of the yolk sac phenotype within the stomach [18]. In our case, given the absence of an adenocarcinomatous component and the absence of risk factors for adenocarcinoma, the gastric YST may have been derived from a germ cell displaced within the wall of the stomach, either from aberrant migration or seeding of circulating germ cells.

Serum AFP and beta‐HCG have emerged as useful markers of specific tumors such as GCTs, with the combined use of these markers being detected in up to 85% of extracranial GCTs in some studies. AFP is a dominant serum protein in embryonic life and is synthesized first by the yolk sac (one of the fetal membranes in utero). After atresia of the yolk sac at 11–12 weeks gestation, the fetal liver becomes the predominant source of AFP. After birth, AFP remains elevated for a few months and then gradually declines, typically reaching adult levels (less than 10 ng/mL) by the end of the first year of life. Elevation in serum AFP beyond the expected age range may indicate underlying conditions such as a liver disorder, conditions that affect the liver, or specific tumors such as hepatoblastoma, YST, or GCTs with a yolk sac component [19]. More than 90% of patients with YSTs have elevated serum AFP levels at the time of diagnosis [20].

Human chorionic gonadotropin (hCG) is a hormone produced by syncytiotrophoblasts, a component of trophoblast tissue which surrounds a growing embryo and eventually forms the placenta after implantation. hCG helps sustain pregnancy and consists of two subunits: the alpha fraction, which is structurally similar to other hormones released by the pituitary gland such as thyroid stimulating hormone (TSH) and luteinizing hormone (LH), and the beta fraction (beta‐hCG), which is unique to hCG and, therefore, is the subunit used to detect pregnancy as early as 2 weeks postconception. Nearly all choriocarcinomas and mixed GCTs with a choriocarcinomatous component produce beta‐hCG. Embryonal carcinoma frequently contains syncytiotrophoblastic giant cells and, as such, is frequently associated with elevated serum beta‐hCG. Rarely has serum beta‐hCG been detected in patients with a YST, typically only if isolated syncytiotrophoblastic giant cells are present (which were not seen in this case). Due to their specificity and sensitivity, serum AFP and beta‐hCG serve as powerful aids in the monitoring for tumor recurrence of GCTs [21].

The medical literature was perused for case reports of gastric YSTs using the search terms “yolk sac tumor” + “stomach” and “endodermal sinus tumor” + “stomach.” Our review found 12 case reports of pure YST reported as gastric primaries (Table 2). Of these 12 case reports, only 1 was in a child, a 3‐year‐old male [28]. Including our case report, 9 gastric YST occurred in males and 4 in females. At the time of presentation, 4 of 13 cases were limited to the stomach. One adult patient had a history of a “partially mature” teratoma without a YST component involving testis and mediastinum 4 years prior to the diagnosis of a gastric pure YST, raising the possibility that the gastric YST may be a recurrence of the prior GCT which due to sampling may not have revealed a YST component [22].

**TABLE 2:** Published case reports of gastric pure yolk sac tumors.

Case reports of gastric pure yolk sac tumors (chronologically)	Age at presentation	Gender	Involvement at presentation	Treatment	Last reported follow‐up
Moller and Raahave [22]	23 years	Male	Stomach, liver^∗^	S	Death following postoperative complications
Zámecník et al. [23]	88 years	Male	Stomach, omentum, lymph nodes	S	Death 4 weeks after surgery
Kanai et al. [24]	87 years	Male	Limited to stomach	S	Death 7 months after surgery
Tahara et al. [25]	74 years	Male	Stomach, liver, lung, lymph nodes	none	Death 6 days after presentation
Kim et al. [12]	61 years	Male	Limited to stomach	S	Alive and w/o recurrence at 3 months after surgery
Magni et al. [11]	62 years	Male	Stomach, lymph nodes	S/C	Death 1 year after surgery
Kunin et al. [26]	61 years	Male	Limited to stomach	S/C	Alive and w/o recurrence at 1 year
Mathew et al. [27]	45 years	Female	Stomach, lymph nodes	S	None provided
Mandelia et al. [28]	3 years	Male	Stomach, liver, omentum	C/S	Alive and w/o recurrence 3 months after C/S
Qureshi et al. [29]	52 years	Male	Stomach, lymph nodes	S/C	Alive with recurrence in transverse mesocolon 16 months after diagnosis
Ibrahim et al. [30]	86 years	Female	Stomach adherent to rectus sheath, small bowel, pancreas, colon	S	Alive 1 week after surgery
Srivastava et al. [31]	43 years	Female	Stomach, liver, lymph nodes	S/C	Death 5 months after surgery
Current case	23 months	Female	Limited to stomach	S/C	Alive and w/o recurrence 3 years after surgery

*Note:* S: surgical resection without report of chemotherapy. S/C: surgical resection followed by chemotherapy. C/S: chemotherapy followed by surgical resection.

^∗^Clinical history significant for surgical resection at Age 19 of teratoma (“partially mature”) involving testis and mediastinum (2 hilar masses) without YST elements.

The development of YSTs is poorly understood, but it has been postulated that misplaced germ cells during embryonic development play a role [32]. YSTs are more likely to occur in the female adolescent and young adult population, with one multicenter study reporting a median age of 20 years [33]. While most malignant GCTs have excellent prognoses, with survival rates between 87% and 97%, YSTs are known for being markedly aggressive. In particular, they are known for their potential to rapidly invade surrounding organ structures and lymph nodes, leading to many YSTs diagnosed at an advanced stage with a worse prognosis [34].

Pediatric and adult YSTs are distinct entities. As a result, in 2016, the World Health Organization divided the GCT category into prepubertal GCTs and postpubertal GCTs [35]. Prepubertal YSTs more often occur in the pure form, and the expected age group of this tumor includes infants and young children, similar to our patient [5]. In contrast, postpubertal YSTs most often occur in the mixed GCT form. It has been observed that postpubertal pure YSTs, while incredibly rare, have been reported to be quite aggressive [36]. Prepubertal mixed GCTs are also incredibly rare and, if not metastatic, have been suggested to respond well to surgical intervention and/or chemotherapy, depending on the stage at diagnosis [37]. In addition to histology, the location of primary tumor has been reported to inform overall survival rates. Pediatric gonadal YSTs have an excellent prognosis, with testicular YSTs estimated to have a ∼90% 5‐year overall survival rate [20]. Female ovarian YSTs have similar 5‐year overall survival rates across age groups of ∼90%. Pediatric extragonadal YSTs have been reported to have lower 5‐year overall survival rates at ∼79.5% [20].

## 4. Conclusion

This gastric pure YST in a 23‐month‐old female is the first reported case in a pediatric female. A review of the medical literature confirmed that pure YSTs are exceedingly rare in the stomach in both children and adults, with a third occurring in females and a third being limited to the stomach at the time of presentation. This case highlights an exceedingly rare diagnosis as a cause of a common condition encountered by pediatric hematology‐oncology providers. For pediatric pathologists, the workup of the small gastric mass biopsy demonstrates the need for a high level of suspicion for GCTs in atypical locations to the direct judicious use of immunohistochemical stains in evaluating a neoplastic infiltrate in a limited sample. Further, this case details a therapy recommendation of subtotal gastrectomy and adjuvant systemic chemotherapy regimen for an unusual pediatric malignancy.

**Nomenclature**
AFP: Alpha fetoprotein
Beta‐hCG: Beta human chorionic gonadotropin
CT: Computed tomography
EGD: Esophagogastroduodenoscopy
FDG‐PET: Fluorodeoxyglucose positron emission tomography
GCT: Germ cell tumor
MRI: Magnetic resonance imaging
TEC: Transient erythroblastopenia of childhood
YST: Yolk sac tumor

## Funding

All authors listed have no funding sources or sponsorship to report as it pertains to this article.

## Disclosure

This case report was previously presented at the 2024 United States and Canadian Academy of Pathology Annual meeting by Sylvestre et al. and has also been published as a preprint by Saleem et al. [38, 39].

## Consent

Written informed consent was obtained from the patient’s mother in accordance with CARE case report guidelines.

## Conflicts of Interest

The authors declare no conflicts of interest.

## Data Availability

The data that support the findings of this study are available from the corresponding author upon reasonable request. The data are not publicly available due to privacy or ethical restrictions.
